# Characterization of Programmed Death-1 Homologue-1 (PD-1H) Expression and Function in Normal and HIV Infected Individuals

**DOI:** 10.1371/journal.pone.0109103

**Published:** 2014-10-03

**Authors:** Preeti Bharaj, Harendra Singh Chahar, Ogechika K. Alozie, Lizette Rodarte, Anju Bansal, Paul A. Goepfert, Alok Dwivedi, N. Manjunath, Premlata Shankar

**Affiliations:** 1 Center of Excellence for Infectious Diseases, Department of Biomedical Sciences, Paul L. Foster School of Medicine, Texas Tech University Health Sciences Center, El Paso, TX, United States of America; 2 Department of Internal Medicine, Paul L Foster School of Medicine, Texas Tech University Health Sciences Center, El Paso, TX, United States of America; 3 Department of Medicine, University of Alabama at Birmingham, Birmingham, Alabama, United States of America; 4 Division of Biostatistics and Epidemiology, Department of Biomedical Sciences, Paul L Foster School of Medicine, Texas Tech University Health Sciences Center, El Paso, TX, United States of America; INSERMU1138, France

## Abstract

Chronic immune activation that persists despite anti-retroviral therapy (ART) is the strongest predictor of disease progression in HIV infection. Monocyte/macrophages in HIV-infected individuals are known to spontaneously secrete cytokines, although neither the mechanism nor the molecules involved are known. Here we show that overexpression of the newly described co-stimulatory molecule, PD1 homologue (PD-1H) in human monocyte/macrophages is sufficient to induce spontaneous secretion of multiple cytokines. The process requires signaling via PD-1H as cytokine secretion could be abrogated by deletion of the cytoplasmic domain. Such overexpression of PD-1H, associated with spontaneous cytokine expression is seen in monocytes from chronically HIV-infected individuals and this correlates with immune activation and CD4 depletion, but not viral load. Moreover, antigen presentation by PD-1H-overexpressing monocytes results in enhanced cytokine secretion by HIV-specific T cells. These results suggest that PD-1H might play a crucial role in modulating immune activation and immune response in HIV infection.

## Introduction

The Ig superfamily costimulatory and coinhibitory molecules serve as important regulators of immune functions. The best-characterized costimulatory/inhibitory pathways include B7.1 (CD80), B7.2 (CD86)/CD28 or CTLA 4; B7-H2 (ICOS-L, CD275)/ICOS (CD278); and B7-H1 (CD274)/PD1 (CD279) [1]–[7]. Functional delineation of these pathways have led to the development of treatment approaches for several human diseases, including autoimmunity and cancer [6]. Recently a new member of the B7 family, B7-H5 has been shown to interact with CD28H to costimulate human T cells [8]

Another such pathway involving a new B7 family member known as PD1 homologue (PD-1H) or V-domain Ig suppressor of T cell activation (VISTA) has recently been described in mice [9]. This molecule appears to be derived from a different precursor than all other B7 family members [7]. PD-1H is a type I transmembrane protein of 309 amino acids (aa) with homology to both PD-1 and its ligand PD-L1 (∼25% sequence identity) but also has distinct features. Unlike most other B7 family members, it has a single Ig-V domain within the extracellular domain. In addition to two canonical cysteines conserved in all B7 family members, the PD-1H Ig-V domain contains three additional cysteines and also another cysteine in the stalk region that is unique to PD-1H and its orthologs [10]. Additionally, unlike PD-1, the cytoplasmic domain does not contain the immunoreceptor tyrosine-based inhibitory motif (ITIM) or the immunoreceptor tyrosine-based switch motif (ITSM). However, it does contain two potential protein kinase C binding sites as well as proline residues that could function as docking sites, suggesting that PD-1H may potentially function as both a receptor and a ligand. Thus, PD-1H is likely to be functionally distinct from other B7 family members. Published studies characterizing this molecule in mice have reported differing functions. While one study found that VISTA (PD-1H) expression on antigen-presenting cells (APCs) suppresses T cell activation and anti-VISTA mAb treatment exacerbates autoimmune encephalomyelitis (EAE), suggesting an immuno-inhibitory role for the molecule [9], another study found that treatment with mAb abolishes graft-vs-host disease (GVHD), suggesting a costimulatory role [10]. Although a human orthologue exists, its function in human hematopoietic cells has not been clearly elucidated. Chronic immune activation is the strongest predictor of disease progression in HIV infection. Immune activation persists (albeit at a lower level) even in individuals on ART. More importantly, the risk of disease progression is independent of viral load, suggesting that factors other than direct viral replication may be important predictors of mortality and non-HIV morbidities (reviewed in [11]–[14]). A key role for chronic immune activation is also supported by the fact that the natural hosts of simian immunodeficiency virus (SIV) do not show immune activation despite high levels of virus replication and do not develop AIDS-associated immunodeficiency [4]. Conversely, SIV infection of unnatural hosts, such as rhesus macaques, results in a high level of immune activation, CD4 T-cell depletion, and rapid progression to AIDS. Understanding the mechanism of chronic immune activation may therefore help identify novel targets for therapeutic intervention in HIV-infection. Direct effects of viral proteins and nucleic acids, innate and adaptive responses to viral antigens, bystander activation of immune cells, and translocation of microbial TLR ligands from the gut to the systemic circulation are some of the myriad factors hypothesized to cause immune activation [15]. It is noteworthy that production of proinflammatory cytokines appears to be a common downstream effect for all these pathways. Monocytes/macrophages in HIV-infected individuals have been reported to be capable of spontaneous cytokine secretion, but neither the mechanism nor the molecules involved in this process have been delineated [15]–[18].

In this study, we found that PD-1H is expressed by human hematopoietic cells, especially monocytes, and is upregulated by certain cytokines and TLR ligands. Overexpression of PD-1H in monocytes leads to spontaneous multiple cytokine secretion in a process that requires signaling via the cytoplasmic domain of PD-1H. Because our studies determined that high expression of PD-1H in monocytes resulted in spontaneous cytokine secretion and also because such spontaneous cytokine secretion occurs in HIV infection, we further characterized this molecule in PBMCs from HIV seropositive individuals. Our data also show that PD-1H is highly expressed in monocytes from HIV infected subjects that correlates with cytokine mRNA expression and immune activation. Moreover, enforced expression of PD-1H in monocytes enhances their ability to stimulate HIV-specific IFN- γ secretion by T cells.

## Results

### Expression of PD-1H in human tissues and hematopoietic cells

**Figure 1:**
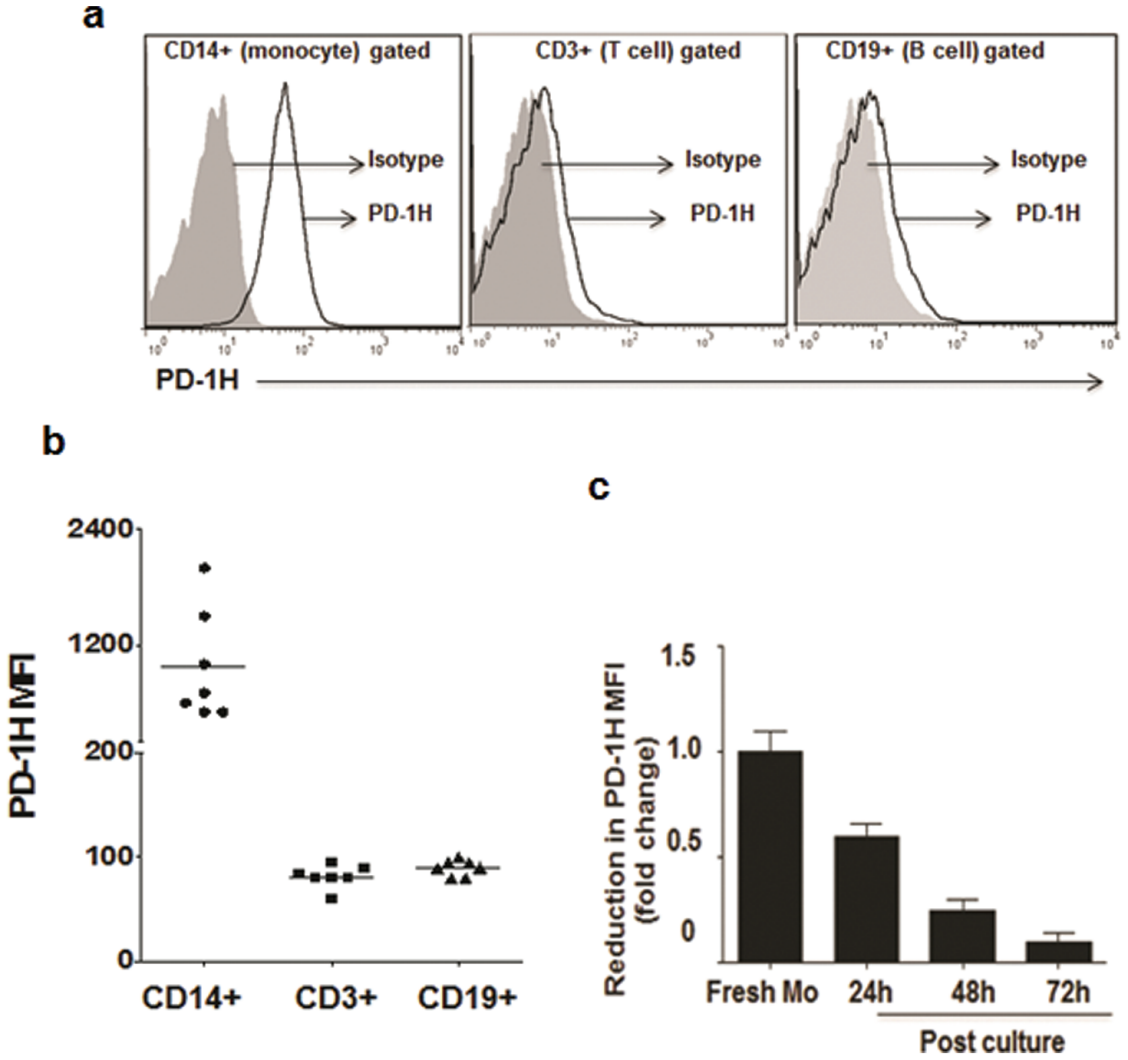
PD-1H expression in subsets of human PBMC. a) PBMCs co-stained for PD-1H along with Monocytes (CD14), T cell (CD3), and B cell (CD19) markers were analyzed by flow cytometry (n = 7). The overlay histogram shows PD-1H expression on CD14, CD3 and CD19 gated cells. The grey filled histogram represents isotype control. b) Cumulative data from seven donors showing PD-1H expression levels expressed as mean fluorescence intensity (MFI). c) Isolated monocytes were cultured for the indicated times and examined for PD-1H expression by flow cytometry (n = 5).

Although an orthologue of mouse PD-1H occurs in humans and an antibody to human PD-1H is available, this molecule remains uncharacterized. Therefore, we first tested PD-1H protein expression in human tissues. Three tissue arrays from different donors were stained with PD-1H antibody and examined by immunohistochemistry. PD-1H was highly expressed in several regions in the brain, thyroid, and stomach. Moderate expression was present in spleen and liver but the expression was quite low in tissues like bone and heart (Fig. S1). Thus, PD-1H is expressed in both hematopoietic and non-hematopoietic cells. Next, we characterized its expression in different subsets of hematopoietic cells. PD-1H protein expression was analyzed by flow cytometry on monocyte- (CD14^+^), T cell- (CD3^+^), and B cell- (CD19^+^) gated populations. The data showed that PD-1H was expressed at high levels on CD14^+^ monocytes but only at low levels on CD3^+^ T lymphocytes, and CD19^+^ B cells (Fig. 1a–b, Fig. S2). We also examined PD-1H expression on monocyte-derived macrophages (MDMs) and monocyte derived dendritic cells (MDDCs) generated by culturing CD14^+^ monocytes for 5 days in the presence of M-CSF or GM-CSF and IL-4 respectively. MDMs and MDDCs expressed low levels of PD-1H compared with freshly isolated CD14^+^ cells (Fig. S3). This difference may have resulted from the prolonged *in vitro* culture involved in their generation. When monocytes isolated by immunomagnetic selection were tested for PD-1H expression at various time points after culture by surface staining for protein, the expression was progressively down-modulated and was barely detectable by 72 h (Fig. 1c). These results are in agreement with studies in mice showing that PD-1H is lost when mouse monocytes are cultured *in vitro* [9] and probably suggest a need for interaction with other cells or the tissue microenvironment to maintain expression.

### Factors that modulate PD-1H expression on monocytes

**Figure 2:**
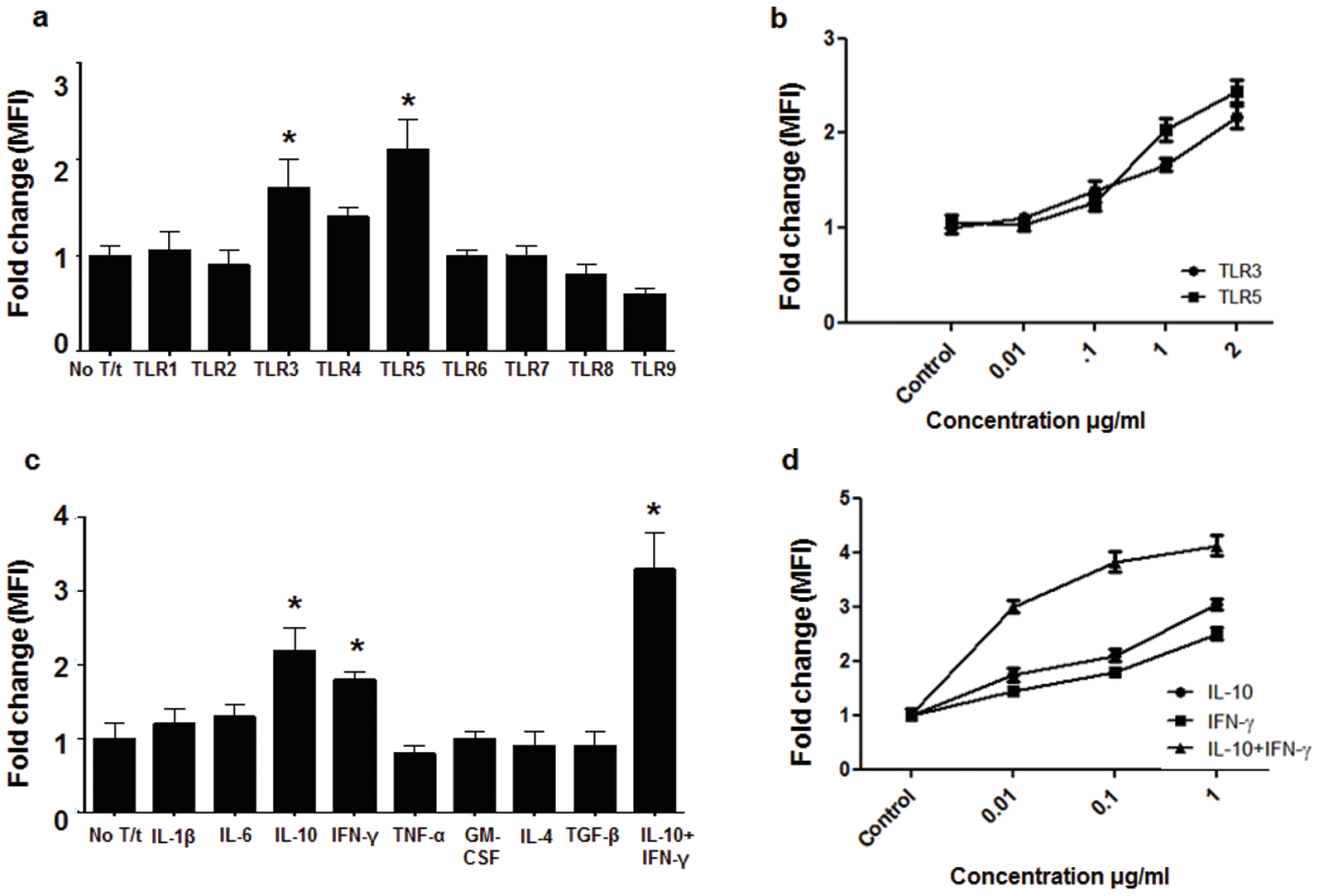
Effect of TLR ligands and cytokines on monocyte PD-1H expression. a) Isolated monocytes were treated with the indicated TLR ligands [TLR1/2: PAM3CSK4 (1 µg/ml), TLR2: HKLM (10^8^ cells/ml), TLR3: Poly I:C (2 µg/ml), TLR4: LPS (200 ng/ml), TLR5: flagellin (1 µg/ml), TLR6/2: FSL1 (0.5 µg/ml), TLR7: Imiquimod (2 µg/ml), TLR8: ssRNA40 (2 µg/ml), TLR9: ODN2006 (5 µM)] or cytokines (c) and examined for PD-1H expression by flow cytometry. The bar graphs and dose response curves represent data (mean±SD) from 3 replicates. * indicates p<0.05 (One way ANOVA followed by Tukey test). b,d) Dose dependent effect of TLRs and cytokines was determined on five different donors. Mean +/- SD for 5 donors is shown.

Since monocytes contribute to the innate immune response, we investigated whether TLR ligands or cytokines influence PD-1H expression. Freshly isolated monocytes were cultured with various TLR ligands (TLR1/2: PAM3CSK4, TLR2: HKLM, TLR3: Poly I:C, TLR4: LPS, TLR5: flagellin, TLR6/2: FSL1, TLR7: Imiquimod, TLR8: ssRNA40, TLR9: ODN2006; Invivogen human TLR agonist kit) and examined for PD-1H expression by flow cytometry. PD-1H expression was significantly upregulated by TLR3 (Poly I:C) and TLR5 (flagellin) agonists, was not affected by TLR1, 2, 4, and was down-modulated by TLR8/9 ligands (Fig. 2a). Engagement of TLR3 as well as TLR5 induced a dose-dependent increase in PD-1H expression (Fig. 2b). The upregulation by TLR3 and TLR5 ligands also occurred when whole PBMCs instead of isolated CD14^+^ cells were tested. These results suggest that PD-1H might be increased following viral (via the dsRNA–TLR3 interaction) and bacterial (via the flagellin–TLR5 interaction) infection.

We also tested whether cytokines regulate PD-1H expression. Isolated monocytes were cultured with IL-6, IL-1β, GM-CSF, IL-4, TGF-β, IL-10, IFN-γ, and TNF-α before testing PD-1H expression by flow cytometry on CD14-gated cells. Significant upregulation was seen with IL-10 and IFN-γ, but not other cytokines (Fig. 2c). Both IL-10 and IFN-γ induced a dose-dependent increase in PD-1H expression (Fig. 2d). A combination of IL-10 and IFN-γ resulted in a synergistic, nearly 4-fold increase in expression. Although the reason for this is unclear, since both of these cytokines are induced after viral infection, it is likely that PD-1H levels go up after infection.

### Overexpression of PD-1H induces spontaneous cytokine secretion by monocytes

**Figure 3:**
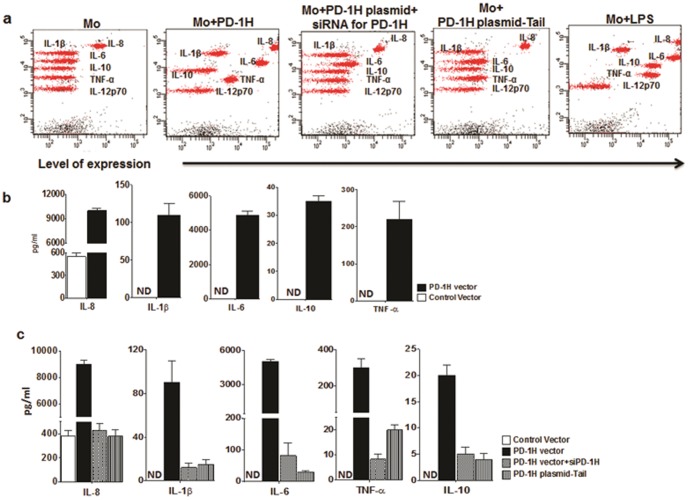
Spontaneous cytokine secretion by monocytes upon overexpression of PD-1H. Monocytes were nucleofected with control or PD-1H expression plasmid without or along with PD-1H siRNA or nucleofected with cytoplasmic domain deficient PD-1H as indicated and the culture supernatants tested for cytokine secretion by cytometric bead array (CBA) or ELISA. a) Representative result with monocytes from one donor (out of 10 donors tested) is shown. b) Supernatants from a) were also tested for individual cytokines by ELISA. Cumulative data from 10 donors are shown c) Cytokine secretion by monocytes in presence of PD-1H, PD-1H lacking cytoplasmic domain, PD-1H with its specific siRNA, vector alone and mock treatment as assessed by ELISA. ND, not detected.

Because monocytes lose PD-1H expression *in vitro*, it was difficult to apply direct approaches to elucidate the function of this molecule. Therefore, we chose to transfect monocytes with a plasmid encoding PD-1H under control of the CMV promoter (this plasmid also has a GFP marker). We nucleofected PD-1H-overexpressing plasmid or a control plasmid into immunomagnetically isolated normal donor monocytes and determined PD-1H levels after 24 h. As shown in Fig. S4a, although transfection with both plasmids was efficient, as assessed by GFP expression (∼60%), PD-1H expression increased only with PD-1H but not control plasmid transfection (Fig. S4b). To test the effect of overexpression, we measured cytokine levels in supernatants after 12, 24, and 48 h using a cytometric bead array (CBA). There was a basal level of IL-8 secretion that was seen in the supernatants from untreated monocyte cultures, consistent with what is reported in the literature [19]. However, compared with controls, PD-1H-transfected monocytes exhibited a dramatic spontaneous secretion of several cytokines, including IL-6, IL-8, IL-1β, and TNF-α (Fig. 3a). The levels of spontaneously secreted cytokines resembled that in LPS-stimulated monocyte cultures. We also confirmed the results with cytokine-specific ELISA for each cytokine, which revealed detection levels comparable with the CBA (Fig. 3b). The spontaneous cytokine secretion following PD-1H overexpression was observed as early as 12 h and continued to increase till 48 h. To determine whether the increased cytokine secretion was specific to PD-1H expression, monocytes were transfected with PD-1H-specific siRNA (validated in cell culture, Fig. S4c) or scrambled siRNA as control along with the PD-1H-encoding plasmid. While scrambled siRNA-treated cells continued to secrete cytokines spontaneously, PD-1H siRNA treatment abolished the response (Fig. 3a, c). We also tested whether PD-1H signalling is needed for cytokine secretion. For this, we transfected monocytes with full-length or cytoplasmic domain truncated PD-1H-expression plasmids. Cytokine secretion seen in full-length PD-1H-transfected cells was abolished when the cytoplasmic domain was deleted (Fig. 3a, c) despite equivalent surface expression of PD-1H, as assessed by antibody staining (Fig. S4d). Collectively, these results suggest that signaling through PD-1H is sufficient to spontaneously induce a cytokine secretion pattern resembling that of fully activated monocytes. Activation status was also reflected by the enhanced HLA-DR expression and phagocytic ability of PD-1H-transfected monocytes (Fig. S5a–b).

### PD-1H is upregulated on monocytes of HIV-infected individuals, and the expression correlates with immune activation

**Figure 4:**
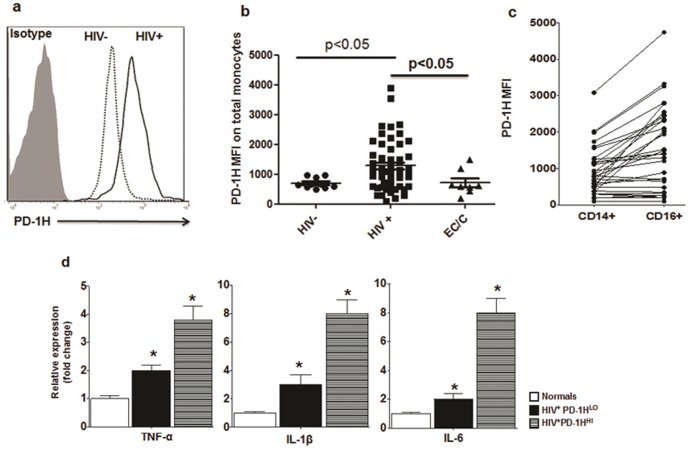
Overexpression of PD-1H on monocytes in HIV infection. A representative histogram (a) and cumulative data (b) of PD-1H expression on CD14 gated PBMC from normal and HIV-infected donors. MFI, mean fluorescence intensity. EC, elite controllers. C, controllers. * indicates p<0.05, Kruskal Wallis test followed by Dunn multiple comparison tests. c) PD-1H expression on CD14+ (classic) and CD16^+^ (activated) monocytes from HIV^+^ donors. Each horizontal line represents expression on CD14+ and CD16+ monocytes from the same individual. d) The mRNA expression level for indicated cytokines in *ex vivo*-isolated monocytes from normal and HIV-infected individuals was assessed by real time PCR. Mean + SD from PBMCs from five donors. (PD-1H^HI^ MFI: 1600-4500; PD-1H^LO^ MFI 700-1500). *  =  p<0.05 (One way ANOVA followed by Tukey test).

Our results in normal human donors suggested that high expression of PD-1H in monocytes leads to spontaneous cytokine secretion. Such spontaneous cytokine secretion also occurs in HIV infection. Therefore, we further characterized this molecule in PBMCs from HIV seropositive individuals. HIV infection is characterized by activation of all immune cell types as well as increased serum cytokine levels. Thus, we tested whether PD-1H expression is increased on monocytes in HIV-infected individuals compared to seronegative controls. We stained PBMCs from HIV-infected patients with PD-1H antibody and examined its expression on CD14-gated cells, using similar staining of normal human PBMCs as a control. We observed that compared to seronegative controls, PD-1H expression on monocytes was two- to four-fold higher in chronically HIV infected subjects, regardless of treatment status (Fig. 4a, b and Fig. S6. PD-1H MFI in normal donors was 880±120 whereas in HIV donors it was 1700±250 (p<0.05, Pearsons two tailed analysis). From 8 elite controllers/controllers tested, PD-1H expression was found to be high in 2 donors whereas in 6 donors, PD-1H level was similar to seronegative subjects.

Monocytes are divided into classical (CD14^++^CD16^−^) and activated (CD14^++^CD16^+^) monocytes [20]. Activated monocytes (5% of the monocyte population) secrete high levels of proinflammatory cytokines and possess potent antigen-presentation capability. PD-1H expression was significantly higher on activated monocytes than on the classic subset (Fig. 4c, Fig. S6). The expression levels were equivalent to that achieved in normal monocytes after transfection with PD-1H plasmid, shown in Fig. S4b. Hence, this level of PD-1H should be sufficient to induce spontaneous cytokine secretion. However, although PD-1H expression was high on activated monocytes that are considered inflammatory and more mature than the other cell subsets, it was difficult to evaluate how this affects cytokine expression at the protein level, because purified CD14^+^ cells rapidly lose PD-1H expression during *in vitro* culture. Thus, we tested cytokine production in freshly isolated monocytes at the mRNA level by real time PCR. RNA isolated from CD14^+^ cells from HIV-infected (PD-1H^HI^ and PD-1H^LO^) and seronegative individuals were tested for mRNA expression levels of TNF-α, IL-1β, and IL-6. Monocytes from HIV-infected patients showed significantly increased levels of TNF-α, IL-1β, and IL-6 compared with seronegative donors (Fig. 4d). We also determined whether the cytokine expression levels in HIV-infected individuals correlated with the intensity of PD-1H expression. Indeed, evaluation of cytokine mRNA levels revealed a significant correlation between levels of PD-1H expression and TNF-α, IL-1β, and IL-6 mRNA levels. We also attempted to discern the cause of PD-1H overexpression in HIV-infected individuals. Because our results in Fig. 2 suggest that IL-10 and IFN-γ can induce overexpression, and also because these cytokines are elevated in HIV infection, we tested whether serum IL-10 and IFN-γ levels correlate with PD-1H expression levels in monocytes. Indeed, PD1H levels correlated well with serum IL-10 and IFN-γ levels (Fig. S7 a-b). Moreover, cultures of monocytes from seronegative individuals in the presence of 20% sera from HIV-infected individuals also resulted in overexpression of PD-1H (Fig. S7c). Thus, HIV-induced cytokines such as IL-10/IFN-γ in serum may be the likely cause of PD-1H upregulation in this setting.

**Figure 5:**
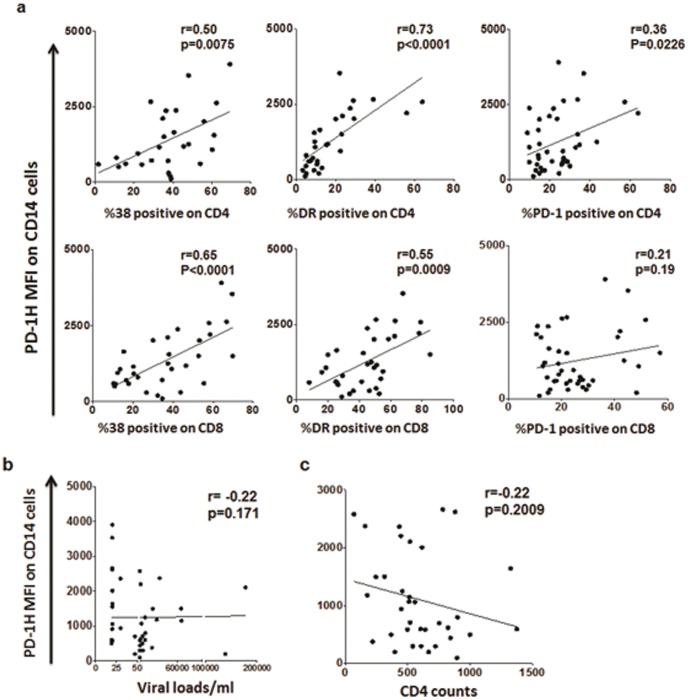
The level of PD-1H expression on monocytes in HIV-infected individuals correlates with immune activation and CD4 T cell depletion, but not viral load. Correlation of PD-1H expression on monocytes with (a) immune activation/exhaustion markers CD38, PD-1 and HLA-DR on CD4 and CD8 T cell subsets (b) viral loads and (c) CD4 counts. (n = 35). r and p values determined by Spearmans non parametric test values. p<0.05 were considered significant.

Next, we tested whether PD-1H expression is associated with markers of immune activation/exhaustion. PD-1H expression on monocytes from HIV infected patients significantly correlated with CD38 and HLA-DR on both CD4 and CD8 T cell subsets (Fig. 5a). On the other hand, the activation/exhaustion marker PD-1 showed good correlation with PD-1H levels on monocytes for CD4 T cells whereas there was only a trend towards significance for CD8 T cells. Elite controllers/controllers showed very low expression of all immune activation markers that corresponds to the low intensity of monocyte PD-1H expression observed in this group.

We also assessed the relationship between PD-1H expression, plasma viral load and CD4 T-cell counts—the latter two being predictors of HIV disease progression. There was no correlation between viral loads and PD-1H expression (Fig. 5b). However, there was a strong trend towards inverse correlation between PD-1H levels and absolute CD4 counts (Fig. 5c).

### PD-1H overexpression enhances monocyte's ability to stimulate HIV-specific IFN-γ production by T cells

**Figure 6:**
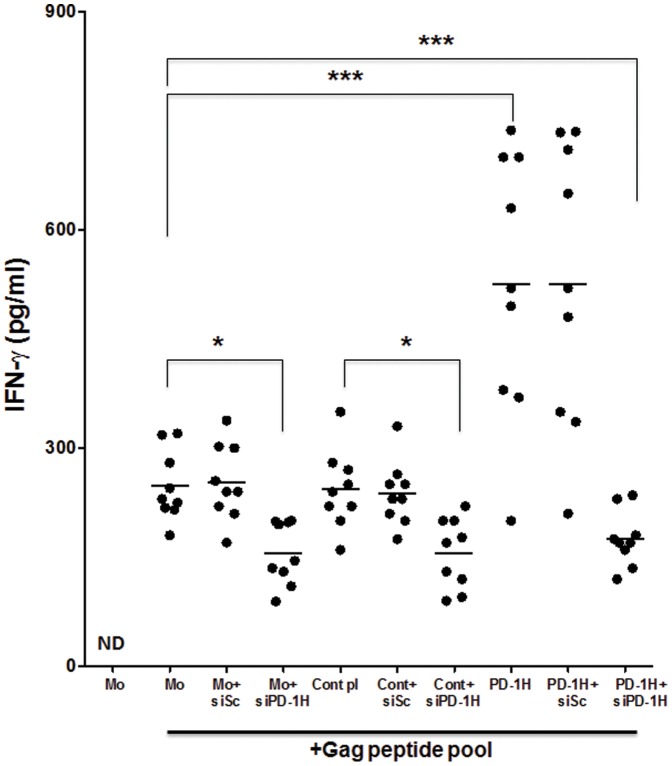
Stimulation with PD-1H- overexpressing monocytes enhances HIV gag-specific IFN-γ response by T cells. Monocytes from HIV-infected individuals were untreated or nucleofected with control plasmid or PD-1H expression plasmid in presence of scrambled siRNA or PD-1H siRNA. After 24 h of transfection, the monocytes were pulsed with HIV clade B gag peptide pool, and used to stimulate autologous T cells. After overnight stimulation, IFN-γ secretion was measured by ELISA. Each symbol represents an individual HIV donor. Mo  =  monocytes. No pep  =  stimulation with monocytes without peptide. ND =  not detected. siSc  =  transfection with control scrambled siRNA; siPD-1H  =  transfection with PD-1H siRNA. Control pl  =  transfection with control plasmid; PD-1H pl  =  transfection with PD-1H expression plasmid.

We tested the impact of PD-1H overexpression on the ability of monocytes to stimulate HIV-specific T cells. Monocytes isolated from HIV-infected individuals were transfected with PD-1H expression plasmid, pulsed with HIV gag peptide pool, and used to stimulate autologous T cells. In parallel, monocytes were similarly transfected with siRNA to silence PD-1H expression. Compared with mock and vector controls, IFN-γ secretion was significantly enhanced when monocytes overexpressing PD-1H were used to stimulate T cells from HIV-seropositive donors (Fig. 6). Consistent with these results, silencing of PD-1H showed the opposite effect of significantly reducing HIV gag-specific IFN-γ production by T cells. Of note, although the most dramatic effect of siRNA treatment on IFN-γ secretion was seen upon co-transfection with PD-1H plasmid, siRNA also reduced IFN-γ production by T cells stimulated with mock and control vector-transfected monocytes, suggesting that PD-1H expression already present on monocytes or induced after antigen exposure is important for their T cell stimulatory function.

## Discussion

This is the first report on the characterization of PD-1H function in human monocytes. We show here that PD-1H acts both as a signaling molecule to induce cytokine secretion by monocytes and as a ligand to stimulate antigen-specific T cell responses. Monocytes from HIV-infected individuals show increased expression of PD-1H, which correlated with expression of immune-activation markers, CD4 depletion and cytokine mRNA levels. Moreover, antigen presentation by PD-1H overexpressing monocytes leads to enhanced HIV-specific T cell responses. Thus, PD-1H regulates both innate and adaptive immunity that could impact HIV-induced immune activation and T cell response.

PD-1H is a newly discovered member of the B7 family of costimulatory/coinhibitory receptors related to PDL-1 and PD-1 [9], [10]. This molecule appears to be derived from a different precursor than all other B7 family members [7] [10]. The cytoplasmic domain of PD-1H does not contain ITIM or ITSM. However, it does contain two potential protein kinase C binding sites as well as proline residues that could function as docking sites, suggesting that PD-1H may potentially function as both a receptor and a ligand. Thus, PD-1H is likely to be functionally distinct from other B7 family members. Indeed, our results suggest that, unlike any other molecule, mere expression of PD-1H is sufficient to elicit a cytokine response in monocytes.

Transfection of monocytes with PD-1H expression plasmid was enough to induce spontaneous cytokine production that was abrogated with siRNA silencing of PD-1H. Moreover, transfection with cytoplasmic tail-deficient PD-1H was unable to trigger cytokine production. Other measures of monocyte activation, such as phagocytosis and DR expression were also enhanced in presence of PD-1H. Because monocytes needed to be transfected with an expression plasmid to induce cytokine secretion, it appears that a certain threshold level of PD-1H expression is required for PD-1H to induce monocyte activation and spontaneous cytokine secretion. That certain cytokines and TLR ligands can increase PD-1H expression may be a regulatory mechanism to ensure that cytokines are induced only when required, as during infection, while avoiding activation of monocytes in a normal setting. However, in the setting of chronic HIV infection, this can also set up a positive feedback loop, with cytokines increasing PD-1H levels, which in turn leads to more cytokine secretion. Several mechanisms, including direct effects of viral proteins and nucleic acids, an innate response to antigen, and translocation of microbial TLR ligands from the gut to the systemic circulation, have been thought to be responsible for inducing immune activation in HIV infection [15]. Elevated levels of proinflammatory cytokines appear to be the common result of all of these pathways. In this study, we found that the higher PD-1H levels on monocytes correlate with immune activation. Whether this is indeed the primary cause for immune activation is not clear. However, a number of studies have shown that HIV infection is associated with increased spontaneous cytokine production by monocytes [5], and it appears logical that this aberrant cytokine secretion contributes to immune activation. It is also possible that a monocyte–T cell interaction may contribute to upregulate immune activation markers on T cells. More studies are needed to directly test the role of PD-1H in HIV-induced immune activation.

Studies in mice suggest contradictory functions for PD-1H, with one study suggesting a stimulatory role [10] while others suggest a predominantly inhibitory role [9], [21], [22]. The reason for this seeming discrepancy is not clear. It is possible that the interpretation could vary depending on whether PD-1H serves as a receptor or a ligand for the particular function studied. Our studies in humans suggest that PD-1H could act both act as a signaling receptor and as a ligand to activate monocytes and enhance T cell responses. This is in agreement with the postulation by Flies et al. on PD-1H potentially functioning both as a receptor and a ligand. We observed that PD-1H expression beyond a certain threshold is sufficient to activate monocytes, leading to spontaneous cytokine production. In this setting, signaling via PD-1H appears to be required since cytokine secretion is abrogated upon deletion of the cytoplasmic domain. Although the exact signaling events remain to be characterized, it is likely that it is indirect because the cytoplasmic domain has only docking modules and lacks ITAMs, ITIMS or ITSMs. Since PD-1H has been reported to activate MMP2, it is also possible that the PD-1H related activation may be an indirect result of increased activity of matrix metalloproteases [23].

Antigen presentation by PD-1H overexpressing monocytes also enhanced HIV gag specific T cell response. In contrast to our results in human cells, the antigen-presentation function was reportedly reduced when PD-1H was overexpressed on antigen-presenting mouse cells [9]. Species-specific differences might account for this discrepancy. Indeed, there are precedents for this: for example, 2B4 acts as an NK-activating receptor in humans but as an inhibitory receptor in mice [24]. There are also differences between mouse and human TLRs and their responsiveness to agonist ligands [2]. These differences highlight the danger of drawing inferences about humans that are based entirely on mouse studies.

In summary, our results suggest that PD-1H in humans is a new costimulatory molecule that regulates both innate and adaptive immunity and that it could play an important role in HIV-induced immune activation and T cell response.

## Materials and Methods

### Human subjects

HIV^+^ and HIV^-^ donors were recruited for the study with the approval of the Institutional Review Board of the Paul L Foster School of Medicine. Peripheral blood mononuclear cells (PBMCs) were isolated by Ficoll Hypaque separation from approximately 70 ml of blood drawn with signed informed consent. Descriptive statistics for the study population is provided in Table 1. Frozen PBMC samples were also obtained from the Department of Medicine at University of Alabama at Birmingham.

**Table 1:** Descriptive statistics for study population.

Characteristic	HIV negative (n = 10)	HIV-1 seropositive (n = 55)	Elite controllers/controllers (n = 8)
Age, median (years, range)	36 (24–60)	40 (21–69)	58 (46–67)
Sex (Male/Female)	10 M	44 M/11F	6 M/2F
CD4+ T cell absolute count, median (cells/mm^3^, range)	983 (870–1280)	516 (57–1260)	832 (667–1374)
Viral load, median (copies/ml, range)	NA	14157 (20–212810)	47 (19–706)

NA, not applicable.

Viral load measurement by Roche Amplicor Monitor v1.5.

### Reagents, antibodies, and chemicals

PD-1H antibody for flow cytometry and immunocytochemistry was obtained from R&D Systems (clone 730804). Secondary antibody was obtained from Dako antibodies tagged to PE (R0480). Antibodies conjugated to PE (CD4, CD8), APC (CD14, HLA-DR, PD-1), FITC (CD3, CD16) and V450 (CD38), and the respective isotype controls were obtained from BD Biosciences. The Human TLR Agonist kit was obtained from Invivogen, and recombinant human cytokines were purchased from R&D Systems. Mission siRNA Universal negative control siRNA (SIC001) and PD-1H siRNAs were purchased from Sigma-Aldrich. The sequences of PD-1H siRNA used are sense: CUCUUGGGCCCUGUGGACA[dT][dT] and antisense UGUCCACAGGGCCCAAGAG[dT][dT]

### PD-1H expression plasmids

The full-length ORF of the PD-1H gene (NM_022153.1) was amplified using cDNA from normal donor monocytes as template and cloned in-frame into a GFP-expressing pReceiver-M03 vector using the primers mentioned above. Empty vector was used as negative control. PD-1H lacking the cytoplasmic domain was generated with primers designed to exclude the 97 aa C-terminal region.

### Immunohistochemistry

Immunohistochemical staining for PD-1H was done on paraffin-embedded normal human tissue arrays (Biomax.us, MD), according to the manufacturer's instructions. Isotype antibody used for IHC was obtained from BD Biosciences (557351). IHC was performed on BenchMark ULTRA (Ventana). Slides were finally mounted and analyzed on a Nikon light microscope at 4X magnification.

### PBMC subset isolation and flow cytometry

PBMC were isolated using Ficol Hypaque. Monocyte/T/B cell isolations were done using negative isolation kits (EasySep human monocyte isolation kit without CD16 depletion or T/B cell enrichment kit) as per the manufacturer's instructions. The viability of cells after isolation was ≥96%, and the purity was >92%. Cells were stained with various antibodies and analyzed using a BD FACS Canto II flow cytometer (BD Biosciences).

### Real Time PCR

PD-1H mRNA levels were quantitated by real time PCR using RNA from monocytes/T cells/B cells isolated from PBMCs of normal donor/HIV-infected subjects. RNA was extracted using Qiazol (Life Technologies, USA) followed by cDNA synthesis using the Superscript III kit (Life Technologies, USA) using 100 ng RNA. PD-1H-specific primers were used for real time PCR using SYBR mix (Life Technologies, USA), and data was normalized to the internal housekeeping control human 18 sRNA. PD-1H mRNA levels were examined using forward primer GGCACGATGTGACCTTCTAC and reverse primer CATGGTGATGGAGAAGTTGC; 18sRNA was detected using GAGAAGACGGTCGAACTTGACT and ACCTACGGAAACCTTGTTACGA as forward and reverse primers. Published primers were used for detection of IL-6, IL-1β, and TNF-α^14^.

### TLR and cytokine treatment

Whole PBMCs or negatively isolated total monocytes were cultured with TLR ligands or cytokines at different concentrations for 24 h before examining for PD-1H expression.

### Plasmid and siRNA nucleofection

Monocytes were isolated from PBMCs of normal or HIV-infected patients by negative isolation, as described earlier, and transfected using the Amaxa Lonza 4D nucleofector system as per the manufacturer's instructions using the Human monocyte cell type transfection protocol. Nucleofection was done with no plasmid (mock) or with control or PD-1H expression plasmid (10 µg plasmid/10^6^ monocytes). In some experiments, scrambled control or PD-1H siRNA (1 µg siRNA for 10^6^ monocytes) was also nucleofected with or without plasmids. After nucleofection, monocytes were transferred to pre-warmed media in a 96-well plate in triplicate for each set and cultured overnight. On the next day, supernatants were harvested for cytokine determination by cytometric bead array (CBA) or ELISA. In some experiments, nucleofected cells were used for antigen stimulation.

### CBA

Proinflammatory cytokines were measured in the supernatants of nucleofected and non-nucleofected monocytes using the BD Cytometric Bead Array (CBA) as per the manufacturer's instructions.

### ELISA

IL-10 was detected using the Quantikine kit from R&D systems. TNF-α, IL-1β, IL-6, and IFN-γ were detected using the ELISA Max Deluxe kits from Biolegend (CA). All the ELISAs were done according to the manufacturer's instructions for quantification in cell culture supernatants or in plasma from HIV-infected patients.

### T cell stimulation

Monocytes (mock, PD-1H-transfected with or without treatment with control or PD-1H siRNA) were pulsed with 5 µg/ml of the HIV Clade B gag peptide pool (obtained from NIH AIDS reagent Program) and co-cultured with autologous T cells overnight at a 1∶10 ratio and supernatants tested for IFN-γ by ELISA.

### Statistical analysis

Statistical analysis was performed with GraphPad Prism software 5.0d (GraphPad Software, Inc., San Diego, CA) or Microsoft Excel. Results are given as means with standard deviations or medians with ranges. One way repeated measures analysis of variance was used to determine the dose effect of various cytokines and TLR ligands on PD-1H expression followed by Tukey's multiple comparison tests. PD-1H was compared among three (normal, HIV+ and ECC) groups using a Kruskal Wallis test followed by Dunn multiple comparison tests. Correlation analysis between PD-1H and T cell activation/exhaustion markers was carried out using Spearman rank correlation (SRC) coefficients. Scatter diagrams were constructed to demonstrate significant correlations between PD-1H and different markers for the HIV cohort data. A linear regression line was plotted for all scatter diagrams. p-values<0.05 was regarded as significant.

## Supporting Information

Figure S1 **Tissue array based immunohistochemical analysis of PD-1H expression in normal donors.** PD-1H protein expression was determined by immunohistochemistry (IHC) on a paraffin-embedded normal human tissue array. Magnification: 4X. An IHC score was assigned to each case according to the following criteria: 3+, intense, cytoplasmic and/or granular staining; 2+, moderate, smooth cytoplasmic staining; 1+, faint cytoplasmic staining. All figures had image captured with an Aperio ScanScope XT (Aperio Technologies, Vista, CA, USA).
(TIF)

Figure S2 **Gating strategy to determine PD-1H expression on CD14+, CD3+ and CD19+ cells.** Human PBMCS were stained with isotype control antibody or PD-1H antibody together with CD14, CD3 and CD19 antibodies. Overlay histograms were derived with FlowJo software after analyzing isotype or PDH1 staining on CD14, CD3 and CD19 gated cells.
(TIF)

Figure S3 **PD-1H expression on cultured macrophages or dendritic cells.** (a) PD-1H expression was determined by flow cytometry on culture derived macrophages or dendritic cells (n = 5). Representative overlay showing data from one of the five independently tested donors.
(TIF)

Figure S4 **Nucleofection with PD-1H expression plasmid induces PD-1H overexpression.** **(a–b)** GFP expression after transfection with control or PD-1H expression plasmid and a representative overlay histogram of PD-1H expression by GFP-gated control or PD-1H plasmid- transfected monocytes are shown. (c) Monocytes were transfected with PD-1H plasmid in the presence of control or PD-1H siRNA and examined for PD-1H expression after 24 h. d) Surface PD-1H expression following nucleofection with full-length or cytoplasmic domain-truncated PD-1H.
(TIF)

Figure S5 **PD-1H overexpression increases HLA-DR expression and phagocytic activity of monocytes.** (a) Monocytes were transfected with control or PD-1H expression plasmid and evaluated for HLA-DR expression by flow cytometry and for phagocytic ability (b) by treating with latex beads coated with phycoerythrin (PE)-labeled rabbit IgG (b). Green, GFP expression by transfected cells. Red, latex uptake by monocytes. Yellow, latex uptake by transfected monocytes.
(TIF)

Figure S6 **Gating strategy to determine PD-1H expression on CD14+, CD16+ and CD3+ cells in HIV+ and HIV- donors.** PBMCs from HIV+ and HIV- donors were stained with antibodies for PD-1H, CD14, CD16 and CD3. Overlay histograms were derived with FlowJo software based on analysis of isotype or PD1H staining on CD14, CD16 and CD3 gated cells.
(TIF)

Figure S7 **PD-1H overexpression in HIV-infected individuals correlate with serum IL-10 and IFNγ levels, and incubation of normal monocytes with sera from HIV patients increases PD-1H expression.** Correlation of PD-1H expression with serum IL-10 (a) or IFNγ **levels** (b) from HIV-infected individuals. Normal PBMCs cultured in the presence or absence of 20% sera from HIV-infected individuals were examined for PD-1H expression after 24 h (c).
(TIF)
