# Supplementary figures and images for: Characterization of Programmed Death-1 Homologue-1 (PD-1H) Expression and Function in Normal and HIV Infected Individuals
Source: PLoS One. 2014 Oct 3;9(10):e109103. doi: 10.1371/journal.pone.0109103 (PMC4184823; doi:10.1371/journal.pone.0109103)

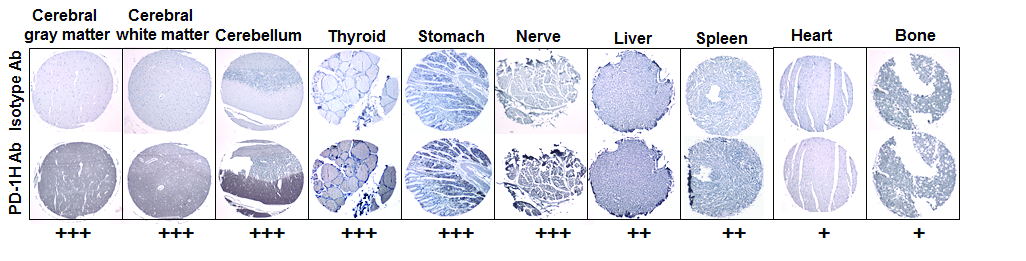

Supplement: Figure S1 — Tissue array based immunohistochemical analysis of PD-1H expression in normal donors. PD-1H protein expression was determined by immunohistochemistry (IHC) on a paraffin-embedded normal human tissue array. Magnification: 4X. An IHC score was assigned to each case according to the following criteria: 3+, intense, cytoplasmic and/or granular staining; 2+, moderate, smooth cytoplasmic staining; 1+, faint cytoplasmic staining. All figures had image captured with an Aperio ScanScope XT (Aperio Technologies, Vista, CA, USA). (TIF) [file pone.0109103.s001.tif]

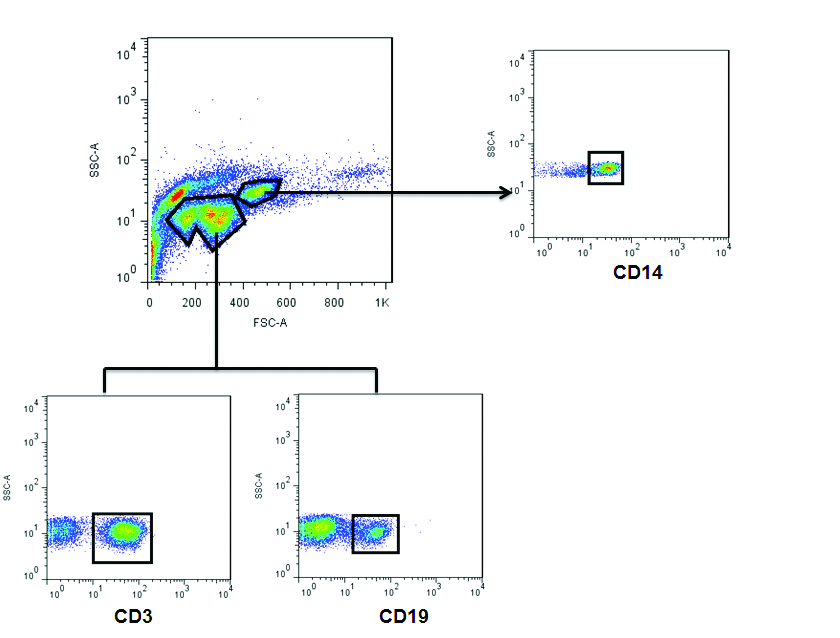

Supplement: Figure S2 — Gating strategy to determine PD-1H expression on CD14+, CD3+ and CD19+ cells. Human PBMCS were stained with isotype control antibody or PD-1H antibody together with CD14, CD3 and CD19 antibodies. Overlay histograms were derived with FlowJo software after analyzing isotype or PDH1 staining on CD14, CD3 and CD19 gated cells. (TIF) [file pone.0109103.s002.tif]

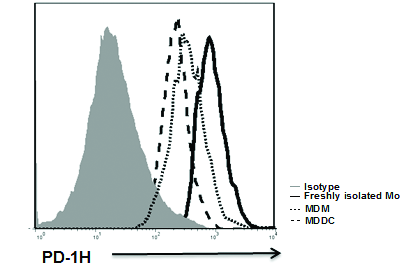

Supplement: Figure S3 — PD-1H expression on cultured macrophages or dendritic cells. (a) PD-1H expression was determined by flow cytometry on culture derived macrophages or dendritic cells (n = 5). Representative overlay showing data from one of the five independently tested donors. (TIF) [file pone.0109103.s003.tif]

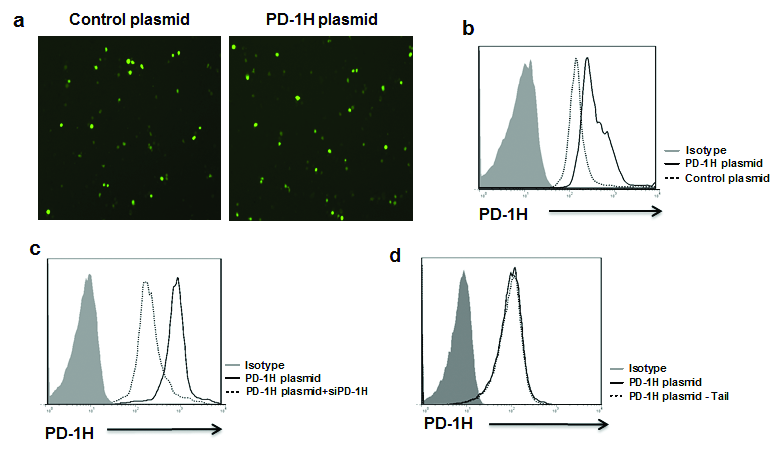

Supplement: Figure S4 — Nucleofection with PD-1H expression plasmid induces PD-1H overexpression. (a–b) GFP expression after transfection with control or PD-1H expression plasmid and a representative overlay histogram of PD-1H expression by GFP-gated control or PD-1H plasmid- transfected monocytes are shown. (c) Monocytes were transfected with PD-1H plasmid in the presence of control or PD-1H siRNA and examined for PD-1H expression after 24 h. d) Surface PD-1H expression following nucleofection with full-length or cytoplasmic domain-truncated PD-1H. (TIF) [file pone.0109103.s004.tif]

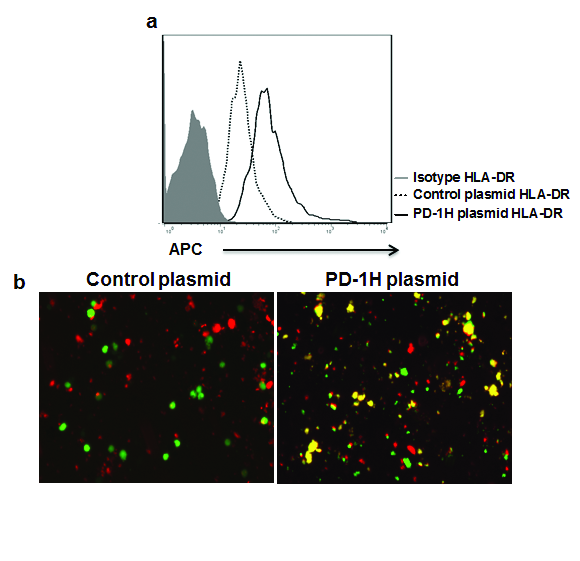

Supplement: Figure S5 — PD-1H overexpression increases HLA-DR expression and phagocytic activity of monocytes. (a) Monocytes were transfected with control or PD-1H expression plasmid and evaluated for HLA-DR expression by flow cytometry and for phagocytic ability (b) by treating with latex beads coated with phycoerythrin (PE)-labeled rabbit IgG (b). Green, GFP expression by transfected cells. Red, latex uptake by monocytes. Yellow, latex uptake by transfected monocytes. (TIF) [file pone.0109103.s005.tif]

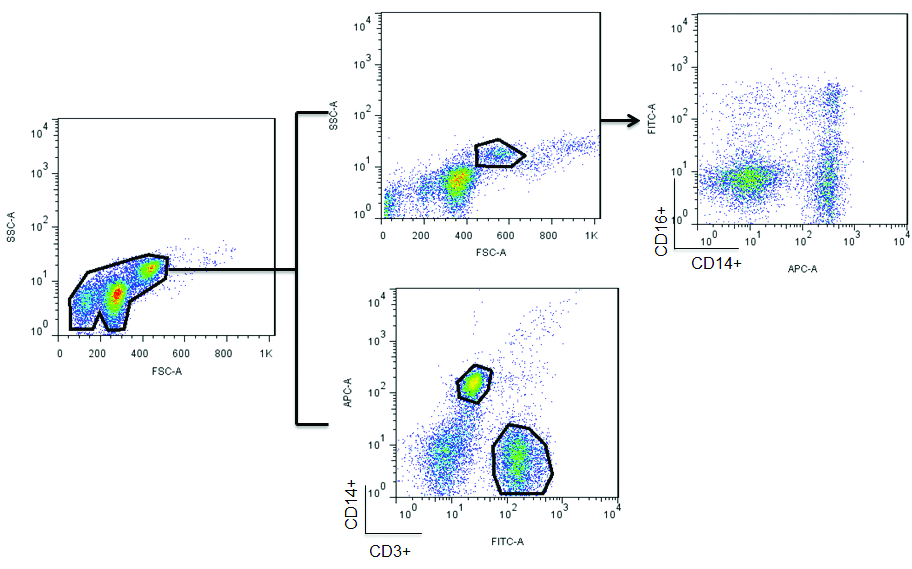

Supplement: Figure S6 — Gating strategy to determine PD-1H expression on CD14+, CD16+ and CD3+ cells in HIV+ and HIV- donors. PBMCs from HIV+ and HIV- donors were stained with antibodies for PD-1H, CD14, CD16 and CD3. Overlay histograms were derived with FlowJo software based on analysis of isotype or PD1H staining on CD14, CD16 and CD3 gated cells. (TIF) [file pone.0109103.s006.tif]

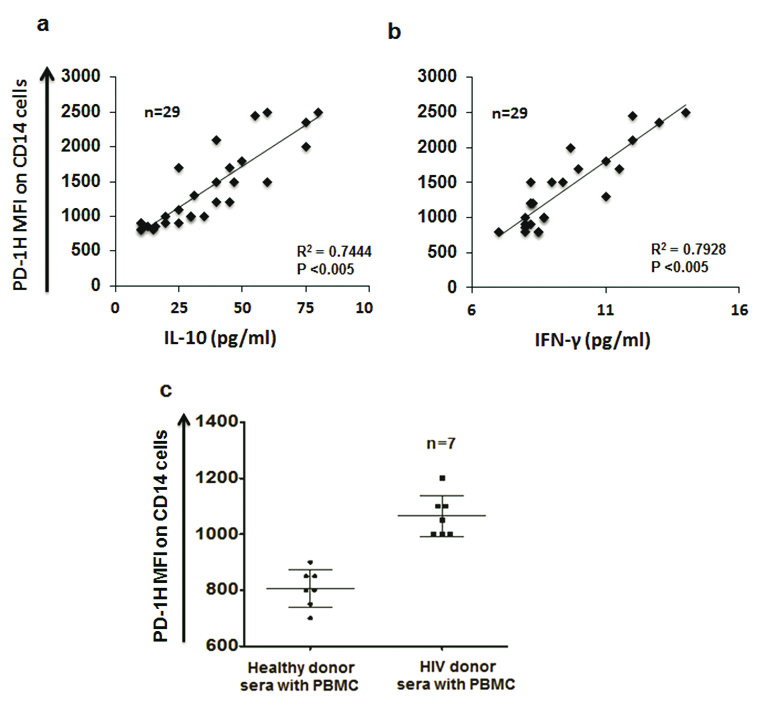

Supplement: Figure S7 — PD-1H overexpression in HIV-infected individuals correlate with serum IL-10 and IFNγ levels, and incubation of normal monocytes with sera from HIV patients increases PD-1H expression. Correlation of PD-1H expression with serum IL-10 (a) or IFNγ levels (b) from HIV-infected individuals. Normal PBMCs cultured in the presence or absence of 20% sera from HIV-infected individuals were examined for PD-1H expression after 24 h (c). (TIF) [file pone.0109103.s007.tif]
